# Embarras de richesses – It is not good to be too anomalous: Accurate structure of selenourea, a chiral crystal of planar molecules

**DOI:** 10.1371/journal.pone.0171740

**Published:** 2017-02-16

**Authors:** Zhipu Luo, Zbigniew Dauter

**Affiliations:** Synchrotron Radiation Research Section, National Cancer Institute, Argonne National Laboratory, Argonne, United States of America; Centro Nacional de Biotecnologia, SPAIN

## Abstract

Selenourea, SeC(NH_2_)_2_, recently found an application as a derivatization reagent providing a significant anomalous diffraction signal used for phasing macromolecular crystal structures. The crystal structure of selenourea itself was solved about 50 years ago, from data recorded on films and evaluated by eye and refined to *R* = 0.15 with errors of bond lengths and angles about 0.1 Å and 6°. In the current work this structure is re-evaluated on the basis of synchrotron data and refined to *R*1 = 0.021 with bond and angle errors about 0.007 Å and 0.5°. The nine planar molecules of selenourea pack either in the *P*3_1_ or in the *P*3_2_ unit cell. All unique molecules are connected by a complex network of Se•••H-N hydrogen bonds and Se•••Se contacts. The packing of selenourea molecules is highly pseudosymmetric, approximating either of the *P*3_1(2)_12, *R*3, and *R*32 space groups. Because the overwhelming majority of diffracted X-ray intensity originates form the anomalously scattering selenium atoms, the measurable anomalous Bijvoet differences are diminished and it was not possible to solve this crystal structure based on the anomalous signal alone.

## Introduction

**Fig 1:**
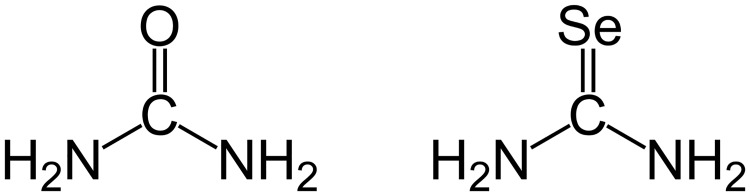
Chemical structures of urea (left) and selenourea (right).

Selenourea, SeC(NH_2_)_2_ (SeUrea) is a small molecule, the selenium derivative of urea (Fig 1), which was the first ever organic compound that was synthesized chemically by Wöhler in 1828 [1]. After several early attempts [2,3], the crystal structure of SeUrea was published almost 50 years ago by Rutherford and Calvo ([4], thereafter R&C), with diffraction data recorded on photographic film using Weissenberg and precession cameras. This structure, containing nine molecules of SeUrea in the asymmetric unit in the space group *P*3_1_, was refined to R1 = 0.120 at the room temperature and R = 0.116 at -100°C. In the latter structure, the estimated standard deviations (esd’s) of bond lengths were about 0.1 Å and esd’s of the bond angles were about 6°.

Our interest in SeUrea was triggered by its use as a convenient reagent for derivatization of macromolecular crystals leading to determination of structures based on the anomalous signal of the incorporated Se atoms [5]. We have obtained accurate diffraction data from SeUrea crystals with the synchrotron radiation and refined the atomic model to estimated bonds and angles accuracy of about 0.007 Å and 0.5°. This accurate model of the SeUrea molecule provides appropriate geometrical targets for refinement of this ligand in complexes with macromolecules, including the hydrogen bonds involving SeUrea.

In addition, the crystal structure of SeUrea presents an interesting and exceptional case from the crystallographic methodology point of view, in the context of its high degree of pseudosymmetry and unusual content of the anomalous signal in diffraction data. These features are discussed and illustrated in detail.

## Methods

The crystalline powder of SeUrea (Sigma-Aldrich) was soaked in 50% (v/v) 2-methyl-2,4-pentanediol (MPD) for 5 min. The remaining undissolved SeUrea crystals were picked up by a nylon loop and vitrified in liquid nitrogen for diffraction data collection. The MPD was used as a cryoprotectant to prevent the ice formation after cooling crystals to 100 K in the stream of cold nitrogen gas. Two data sets from two separate crystals were collected at the wavelength of 0.8 Å on the Rayonix MX300HS detector at the SER-CAT 22ID beam line of the Advanced Photon Source, Argonne National Laboratory, USA. The data were processed by *HKL*2000 [6] without merging the Friedel mates and converted to *SHELX* format by *XPREP* [7]. The crystal structure was solved by *SHELXT* [8] and refined by *SHELXL* [9]. The two crystals turned out to have an opposite chirality, in space groups *P*3_1_ and *P*3_2_. The better refined structure in *P*3_1_ is discussed in the following sections, but the diffraction data and refinement statistics are presented in Table 1 for both crystals. The atomic parameters of the *P*3_1_ structure are listed in Table 2. The bond lengths and angles in all nine independent molecules of SeUrea are listed in Table 3.

**Table 1:** Crystal data and structure refinement for selenourea, SeC(NH_2_)_2_. The Friedel mates were not merged, but treated as separate reflections in all calculations.

Data sets	Crystal 1	Crystal 2
Formula weight	123.02	123.02
Temperature (K)	100	100
Space group	P3_1_	P3_2_
Cell dimensions		
a = b (Å)	15.13	15.13
c (Å)	12.91	12.92
Z	27	27
Crystal size (mm^3^)	0.10 × 0.05 × 0.03	0.25 × 0.20 × 0.20
Radiation wavelength (Å)	0.800	0.800
2Θ range (°)	3.50–55.42	4.98–55.43
Reflections collected	30,492	30,437
Independent reflections	5,592	5,590
R_merge_	0.056	0.092
Data/restraints/parameters	5,592/217/325	5,590/19/325
Goodness-of-fit on F^2^	1.042	1.048
Final R indices [I> = 2σ (I)]	R_1_ = 0.0222, wR_2_ = 0.0607	R_1_ = 0.0315, wR_2_ = 0.0787
Final R indices [all data]	R_1_ = 0.0227, wR_2_ = 0.0609	R_1_ = 0.0319, wR_2_ = 0.0789
Flack parameter	-0.003(6)	0.009(11)
CCDC code number	1521873	1521876

**Table 2:** Atomic coordinates, Ueq values (in Å^2^). The uncertainties (related to the last decimal digit) are in parentheses.

Atom	x	y	z	Ueq
Se_1	-0.03486(4)	-0.06824(4)	0.27914(5)	0.00817(15)
C_1	0.0452(4)	0.0743(4)	0.2769(4)	0.0120(12)
N1_1	0.0567(4)	0.1257(4)	0.1908(4)	0.0199(12)
N2_1	0.0933(4)	0.1257(4)	0.3615(4)	0.0241(12)
Se_2	0.64231(5)	0.26430(4)	0.15816(5)	0.01333(15)
C_2	0.6720(4)	0.3998(4)	0.1619(4)	0.0108(12)
N1_2	0.6613(4)	0.4447(4)	0.0788(4)	0.0186(11)
N2_2	0.7017(4)	0.4528(4)	0.2490(4)	0.0226(12)
Se_3	0.28985(5)	0.60531(4)	0.13361(5)	0.01084(15)
C_3	0.3820(4)	0.7454(4)	0.1339(4)	0.0110(12)
N1_3	0.4090(4)	0.7995(4)	0.0483(4)	0.0173(11)
N2_3	0.4240(4)	0.7924(4)	0.2232(4)	0.0182(11)
Se_4	0.29828(4)	0.35726(4)	0.23551(4)	0.00683(14)
C_4	0.3585(4)	0.2755(4)	0.2253(4)	0.0076(11)
N1_4	0.4147(4)	0.2827(4)	0.1435(3)	0.0137(10)
N2_4	0.3458(4)	0.2093(4)	0.2991(4)	0.0188(11)
Se_5	0.29979(4)	0.26410(5)	0.55963(4)	0.00569(13)
C_5	0.4185(4)	0.3930(4)	0.5505(4)	0.0077(10)
N1_5	0.4473(4)	0.4418(3)	0.4620(4)	0.0162(11)
N2_5	0.4738(3)	0.4360(4)	0.6349(3)	0.0127(10)
Se_6	0.38732(4)	0.35966(4)	0.89154(4)	0.00566(14)
C_6	0.2443(4)	0.2873(4)	0.8962(4)	0.0079(11)
N1_6	0.1906(4)	0.2320(4)	0.8158(3)	0.0125(10)
N2_6	0.1956(4)	0.2916(4)	0.9795(4)	0.0161(11)
Se_7	0.71170(4)	0.68529(4)	0.18036(4)	0.00535(14)
C_7	0.5685(4)	0.6225(4)	0.1820(4)	0.0064(11)
N1_7	0.5141(4)	0.5738(4)	0.0995(3)	0.0144(11)
N2_7	0.5199(4)	0.6252(4)	0.2669(3)	0.0121(10)
Se_8	0.62917(4)	0.69335(4)	0.51909(4)	0.00597(14)
C_8	0.7309(4)	0.6582(4)	0.5228(4)	0.0113(12)
N1_8	0.7765(4)	0.6542(4)	0.4373(3)	0.0140(10)
N2_8	0.7586(4)	0.6367(4)	0.6116(4)	0.0242(12)
Se_9	0.63120(4)	0.60300(4)	0.85061(4)	0.00529(13)
C_9	0.6509(4)	0.7353(4)	0.8606(4)	0.0101(11)
N1_9	0.6339(4)	0.7791(4)	0.7803(3)	0.0151(11)
N2_9	0.6808(4)	0.7864(4)	0.9476(4)	0.0206(11)

**Table 3:** Bonds lengths (Å) and angles (°).

Molecule	Se-C	C-N1	C-N2	Se-C-N1	Se-C-N2	N1-C-N2
1	1.873(6)	1.317(7)	1.327(8)	120.7(4)	120.8(4)	118.5(5)
2	1.867(6)	1.322(7)	1.323(8)	121.3(4)	120.7(4)	118.0(5)
3	1.866(6)	1.313(7)	1.337(7)	122.0(4)	119.5(4)	118.6(5)
4	1.872(6)	1.325(7)	1.326(7)	120.4(4)	121.0(4)	118.6(5)
5	1.882(5)	1.312(7)	1.330(7)	121.0(4)	119.8(4)	119.2(5)
6	1.876(6)	1.326(7)	1.323(7)	120.5(4)	120.4(4)	119.2(5)
7	1.881(6)	1.321(7)	1.331(7)	120.7(4)	120.5(4)	118.7(5)
8	1.863(6)	1.320(7)	1.316(7)	121.2(4)	120.3(4)	118.6(5)
9	1.876(6)	1.323(7)	1.309(7)	120.9(4)	120.8(4)	118.2(5)
Average	1.873(6)	1.322(7)		120.7(5)		118.6(4)

## Structure

**Fig 2:**
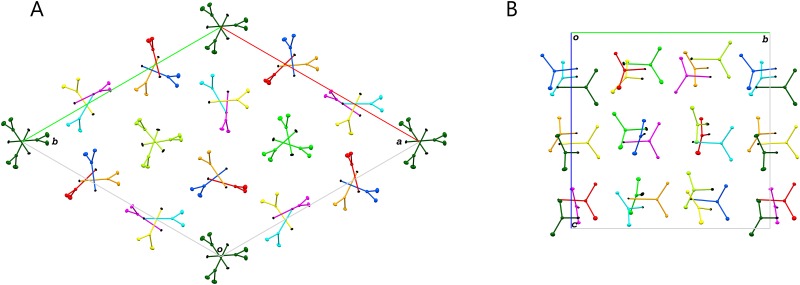
SeUrea crystal packing viewed along c axis (A) and a axis (B) in the unit cell. The Se atoms are colored black and the remaining atoms are colored separately for each group of symmetry equivalent molecules. The atoms are represented as thermal ellipsoids of 50% probability. The Figure was created by Mercury [20].

In general, the structure of SeUrea is isomorphous to that described by R&C. The unit cell of the *P*3_1_ symmetry contains 27 molecules of SeUrea arranged in five helical columns around three non-equivalent crystallographic 3_1_ axes and two similar columns forming the non-crystallographic, approximate 3_1_ axes, as illustrated in Fig 2. The details of this highly pseudosymmetric arrangement are discussed in the next section.

**Fig 3:**
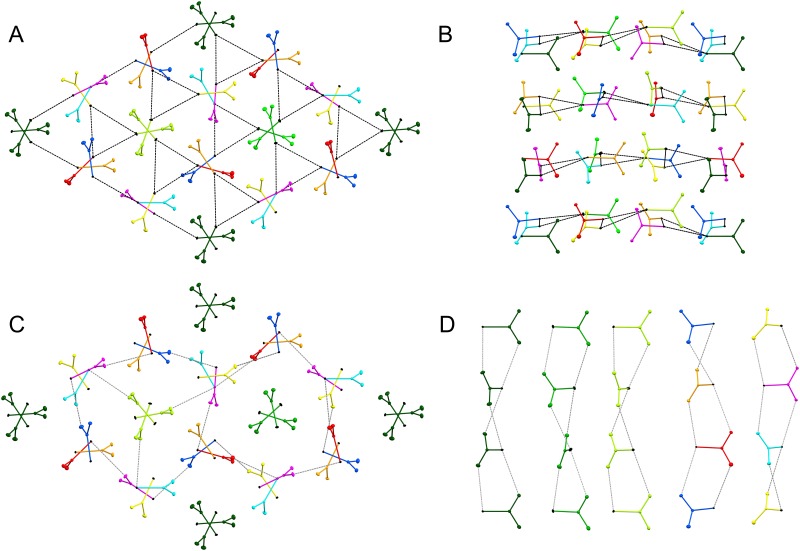
The Se•••Se (black dash) contacts and hydrogen bonds (grey dash) between SeUrea molecules. (A) The Se•••Se contacts between neighboring SeUrea molecules viewed along the c axis. (B) The same contacts viewed along the a axis. (C) H-bonds between SeUrea molecules in the neighboring 3_1_ columns viewed along the c axis. (D) H-bonds between SeUrea molecules within the same 3_1_ columns viewed along the a axis. The three leftmost columns show the crystallographic and the two rightmost columns show the non-crystallographic 3_1_ axes. The Figure was created by Mercury [20].

The van der Waals radius of the Se atom is 1.90 Å [10], but many Se•••Se contacts between neighboring columns are slightly shorter than twice of this value, down to 3.55 Å (Fig 3A and 3B, Table 4). The SeUrea molecules within each 3_1_ column are connected through hydrogen bonds between one of the amide NH_2_ groups of one SeUrea molecule (H-bond donor) and the Se atom of the SeUrea molecule from the next layer (Fig 3C and 3D and Table 5). In addition, the amide groups of SeUrea molecules are engaged in hydrogen bonds with Se atoms form neighboring columns. In effect, all hydrogen atoms of the amide groups serve as donors in hydrogen bonds except for one (on the N1_3 atom, Table 5), and some are engaged in bifurcated H-bonds with two Se acceptors. The donor-acceptor lengths of all H-bonds vary between 3.4 and 3.8 Å, within the range observed also in other small molecule crystal structures containing the selenocarbonyl group [11].

**Table 4:** Intermolecular contacts between Se atoms (Å) comparable with the sum of van der Waals radii (2 x 1.90 Å). The uncertainties of these distances are 0.001 Å.

	Se_1	Se_2	Se_3	Se_4	Se_5	Se_6	Se_7	Ser_8	Se_9
Se_1	-	-	-	-	3.563	-	3.992	-	-
Se_2	-	-	-	-	-	3.668	-	-	3.659
Se_3	-	-	-	4.039	-	-	-	3.548	-
Se_4	-	-	4.039	-	-	-	-	3.705	-
Se_5	3.563	-	-	-	-	-	3.761	-	-
Se_6	-	3.668	-	-	-	-	-	-	3.724
Se_7	3.992	-	-	-	3.761	-	-	-	-
Se_8	-	-	3.548	3.705	-	-	-	-	-
Se_9	-	3.659	-	-	-	3.724	-	-	-

**Table 5:** Hydrogen bond lengths (Å) between Se atoms and amide nitrogen atoms. Hydrogen bonds within the same 3_1_ columns are in bold. The uncertainties of these distances are 0.005 Å.

	Se_1	Se_2	Se_3	Se_4	Se_5	Se_6	Se_7	Se_8	Se_9
N1_1	**3.447**	-	-	3.628	-	-	-	3.655	-
N2_1	**3.474**	-	-	3.697	-	-	-	-	-
N1_2	-	**3.472**	-	-	-	-	3.574	-	-
N2_2	-	**3.454**	-	-	-	-	3.556	-	-
N1_3	-	-	**3.488**	-	-	-	-	-	-
N2_3	-	-	**3.470**	-	-	3.426	-	-	-
N1_4	-	3.597	-	-	-	**3.549**	-	-	3.760
N2_4	-	-	-	-	**3.614**	-	-	-	3.527
N1_5	-	-	-	**3.519**	-	-	-	3.482	-
N2_5	-	-	-	-	-	**3.537**	-	3.711	-
N1_6	3.618	-	-	-	**3.619**	-	-	-	-
N2_6	3.718	-	-	**3.574**	-	-	3.748	-	-
N1_7	-	-	3.682	3.713	-	-	-	-	**3.589**
N2_7	-	-	3.758	3.774	-	-	-	**3.563**	-
N1_8	-	-	-	-	-	3.670	**3.557**	-	-
N2_8	-	-	-	-	-	3.498	-	-	**3.538**
N1_9	-	-	-	-	3.684	-	-	**3.602**	-

The crystal structure of SeUrea is noncentrosymmetric, even though the individual SeUrea molecules are planar and have the mm2 (C_2v_) symmetry. The chirality of the space group *P*3_1_ is therefore a result of specific packing of SeUrea molecules in the crystal. Indeed, the crystal structure of SeUrea described here is in the space group *P*3_1_, which is supported by the Flack parameter [12] refined to -0.003(6), whereas after inversion of atomic coordinates and change of the space group to *P*3_2_ the Flack parameter became 1.013(6). However, the structure of the *P*3_2_ crystal, obtained in exactly same way, refined to R1 = 0.032 with the Flack parameter of 0.009(11) in the space group *P*3_2_ (and of 1.003(11) after inversion to *P*3_1_), confirming that the chirality of the SeUrea crystals is selected at random.

The final model of SeUrea discussed here was refined against the diffraction data in the space group *P*3_1_ with merged Friedel mates.

## Pseudosymmetry

**Fig 4:**
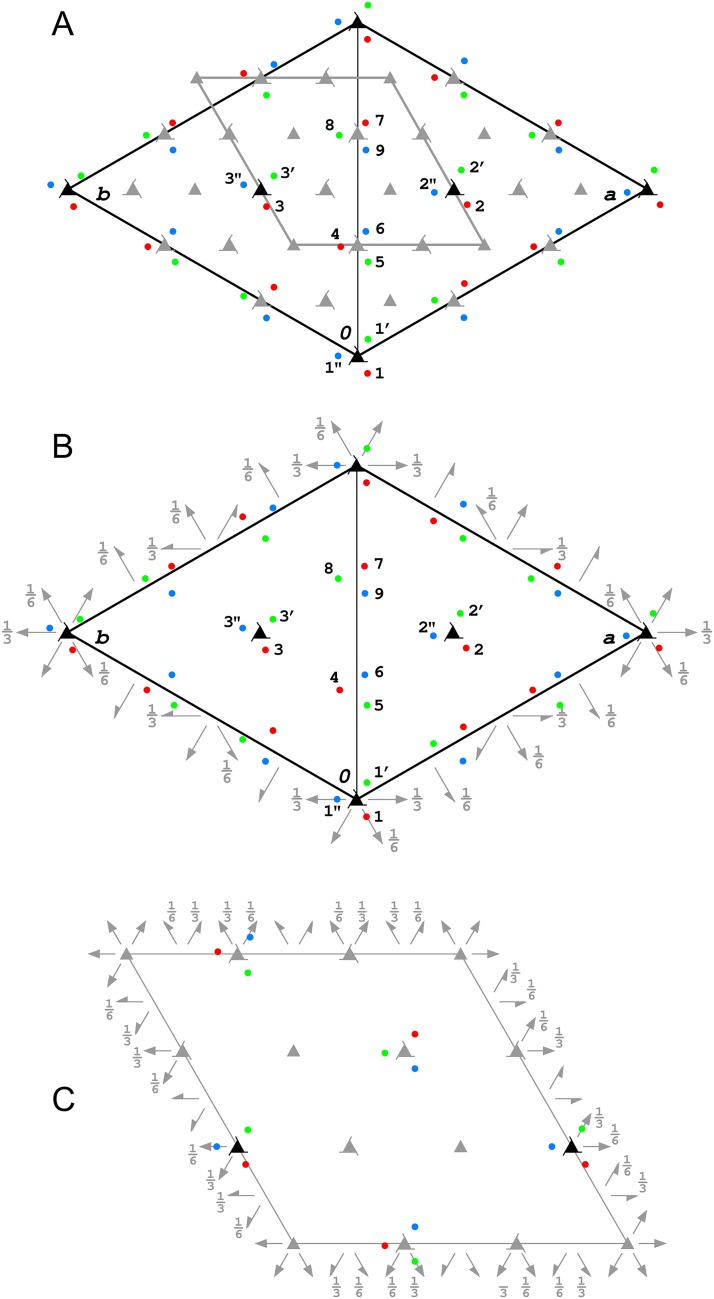
(A) Projection of the SeUrea unit cell along the c-axis with all positions of Se atoms marked in three colors. The red color corresponds to Se atoms within the lowest z-layer, the green color to Se atoms within the layer higher by c/3 and the blue color to Se atoms again higher by c/3. The crystallographic 3_1_ axes are presented in black, the approximate, non-crystallographic 3-fold axes of various kind and the corresponding *R*3 cell are shown in grey. (B) The selenium atom positions presented in the approximate *P*3_1_12 unit cell, with 1.33 Å of the rms difference from ideally symmetric constellation of this space group. The crystallographic 3_1_ axes are in black and the approximate two-fold axes in grey. (C) The idealized approximate symmetry elements of the *R*32 space group relate the positions of selenium atoms within the rms deviation of 1.61 Å.

As pointed out by R&C, the arrangement of 27 SeUrea molecules in the whole unit cell is characterized by a certain degree of pseudosymmetry and this was utilized by them in the original structure solution. Each of the three crystallographic 3_1_ axes at (0,0,z), (1/3,2/3,z) and (2/3,1/3,z) is surrounded by three SeUrea molecules forming right-handed helices. Two triples of SeUrea molecules form very similar helices around approximately (1/3,1/3,z) and (2/3,2/3,z) lines, according to the two additional, approximate (non-crystallographic) 3_1_ axes, Fig 2A. In effect 9 of the total 27 SeUrea molecules in the unit cell are arranged around the crystallographic 3_1_ axes and the remaining 18 molecules are analogously arranged around the non-crystallographic 3_1_ axes. All SeUrea molecules are positioned in three layers with respect to the c-axis with the fractional z coordinates of the Se atoms in the following three ranges: 0.13–0.28, 0.46–0.61, 0.80–0.95, with average values separated by 1/3 of the c cell parameter (Fig 2B). The location of Se atoms in the unit cell is presented in Fig 4A, which shows both crystallographic and approximate, non-crystallographic three-fold axes of the 3, 3_1_, and 3_2_ types. The arrangement of all three kinds of the threefold axes corresponds to the *R*3 space group with three times smaller unit cell with the edges transformed as follows: **a**^R^ = (**a**^P^-**b**^P^)/3, **b**^R^ = (2**a**^P^+**b**^P^)/3, **c**^R^ = **c**^P^.

However, the repetition of Se atom positions in the a,b-plane is more symmetric than along the c direction. Superposition of all five individual helical triples of Se atoms in the a,b-plane (disregarding differences in z coordinates) gives the root-mean-square (rms) deviation from the average position of 0.20 Å. The rms deviation of the Se atoms z-coordinates from their respective average values of the three layers is 0.67 Å. The rms deviation of Se atoms from the ideal *R*3 symmetry is 1.07 Å.

In addition to the arrangement of Se atoms according to the set of the exact and approximate 3-fold axes, they also conform to a number of the approximate 2-fold symmetry axes perpendicular to the c axis, which together with the crystallographic 3_1_ axes form the approximate *P*3_1_12 space group, as illustrated in Fig 4B. The rms deviation of the Se atoms from this idealized arrangement is 1.33 Å. The combination of all approximate 3-fold and 2-fold axes of various types forms the *R*32 space group presented in Fig 4C (within the three times smaller unit cell) where the Se atoms agree with this symmetry within the rms deviation of 1.61 Å.

The pseudosymmetric arrangement of SeUrea molecules in the unit cell consequently modulates the reflection intensities in the reciprocal space, evident in the statistics of the normalized structure factors. The <E^2^> value for all 2797 reflections is 0.993, for 931 reflections with—h+k = 3n is 2.086, and for 1866 reflections with—h+k≠3n is 0.448, clearly identifying the prominence of the smaller cell. The statistics of reflections of the pseudo-rhombohedral cell, with indices transformed accordingly [H = (-h+k)/3, K = (h+2k)/3, L = l] are also indicative for the small cell, with <E^2^> value for all 931 reflections of 0.982, for 309 reflections with—H+K+L = 3n of 2.099, and for 622 reflections with—H+K+L≠3n of 0.428. It may be pointed out that the intensity statistics depend mainly on the positions of the Se atoms, since they are responsible for Z^2^(Se)/[Z^2^(Se)+Z^2^(C)+2 Z^2^(N)+4 Z^2^(H)] = 0.90, i.e. about 90% of the total reflection intensities. All diffraction data merge in the *P*3_1_12 symmetry with the *R*_merge_ = 0.203 and the subset of rhombohedral reflections (H,K,L) merge in the *R*32 symmetry with *R*_merge_ = 0.154, confirming a high degree of pseudosymmetry present in the reciprocal space. No merohedral twinning, theoretically possible in the *P*3_1_ symmetry, was evident in the diffraction data statistics or during refinement of the structure by *SHELXL* [9].

## Anomalous signal in the diffraction data

**Fig 5:**
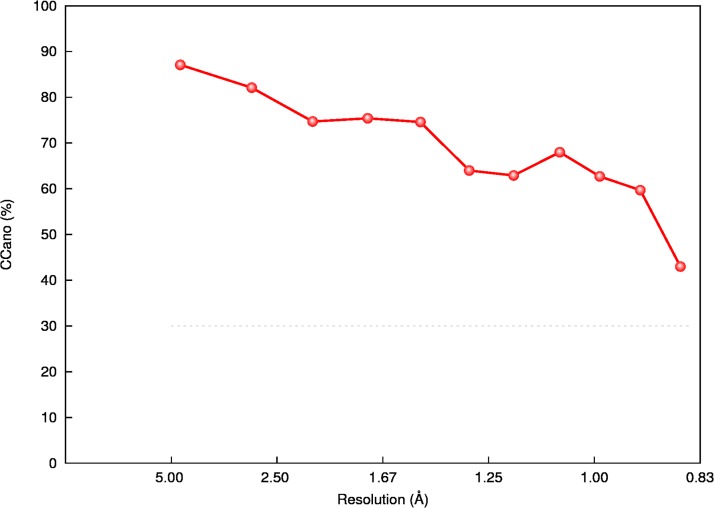
The correlation coefficient between the anomalous differences in the two randomly selected and merged half-data sets (CC_ano_) versus resolution.

At the wavelength of 0.80 Å used for collecting the diffraction data, the anomalous scattering coefficients of Se are: f’ = -0.64 e, f” = 2.72 e. It was therefore expected that a significant anomalous signal would be present in the diffraction data (Fig 5). However, the crystal of SeUrea constitutes an interesting and somewhat complicated case in the context of the anomalous scattering effects.

**Fig 6:**
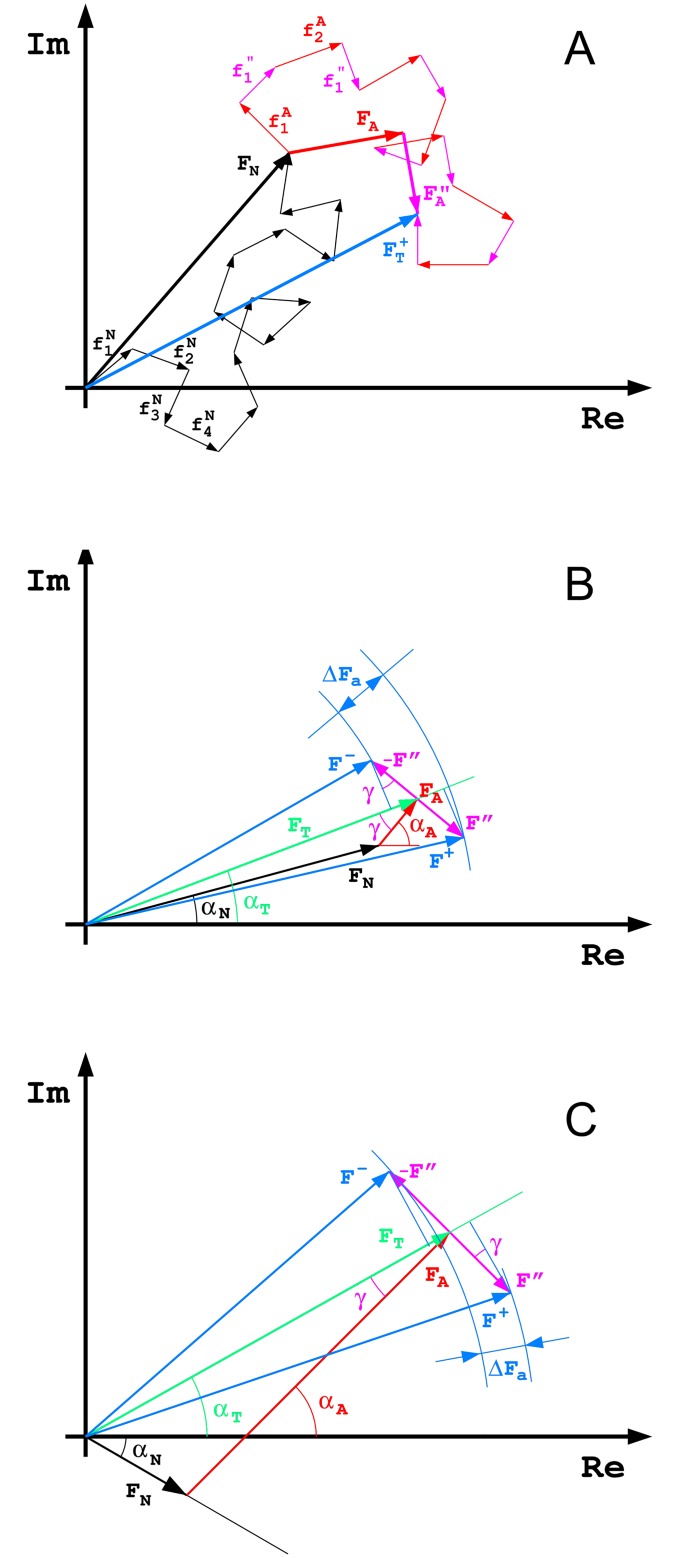
Vector representation of the anomalous scattering effects. (A) The contributions of individual normal and anomalous atoms to the total structure factor. (B) The case of majority of normal atoms and a few anomalous atoms, F_N_ > F_A_. (C) The case with majority of the anomalously scattering atoms F_A_ > F_N_ as in the crystal of SeUrea. See the text for explanation of all vector relations.

If crystal structure consists of a number of “normally” scattering atoms (without any anomalous component) and of several atoms displaying observable anomalous effect, the total structure factor F_T_ of a selected reflection can be presented as a vector sum of contributions from individual atoms:

$${\text{F}}_{\text{T}}=\Sigma_{\text{j}}^{\text{N}}({\text{f}}_{\text{j}})+\Sigma_{\text{j}}^{\text{A}}({\text{f}}_{\text{j}}^{\text{o}}+{\text{f}}_{\text{j}}^{\text{'}}+{\text{if}}_{\text{j}}^{"})={\text{F}}_{\text{N}}+{\text{F}}_{\text{A}}$$

as illustrated in Fig 6A, where black vector F_N_ is the total component of N_N_ normal atoms, the red vector F_A_ represents the normal scattering component of the N_A_ anomalous scatterers (based on the real part of atomic form factors f_j_° + f_j_’), the pink vector F” corresponds to the purely anomalous contribution f” of the N_A_ anomalous scatterers. The direction of the F” vector is always rotated with respect to the direction of the F_A_ vector by -π/2, if all anomalous atoms are of the same kind. The blue F_T_^+^ vector represents the total, observable structure factor of the reflection F(hkl).

If a large number of atoms are spread in the unit cell randomly, as was e.g. assumed in the classic derivation of the Wilson probability distribution of reflection intensities [13] and in many other crystallographic theories, the atomic contributions form a random walk of small vectors in the Argand diagram of Fig 6A. The rms distance after N random unit steps of length d is d √N [14]. Hence, the large vectors in Fig 6A can be approximated as follows: |F_N_| = f_N_ √N_N_, |F_A_| = (f_A_° + f_A_’) √N_A_, |F”| = f_A_” √N_A_.

In the usual practice of phasing macromolecular crystal structures by the Multi- or Single-wavelength Anomalous Diffraction (MAD or SAD, [15]) methods, the great majority of the structure is built by the non-anomalous atoms and the normal contribution of the anomalous atoms is small, a situation which is presented in Fig 6B. The blue vector F^+^ is the total, measurable amplitude of reflection F(hkl), and the vector F^-^ is (the complex conjugate of) its Friedel mate F(-h-k-l) (the original F^-^ reflection has a negative phase and would lie below the abscissa axis). It is evident that the largest measured Bijvoet difference ΔF^±^ occurs when the red vector F_A_ is perpendicular to the total (averaged for both Friedel mates) green vector F_T_, i.e. when |α_A_ − α_N_| = π/2, or sinγ = F_A_/ F_N_. The average measured Bijvoet difference ΔF^±^ corresponds to the average projection of the pink vector 2F” on the direction of F_T_, so that <ΔF^±^> = 4F”/π, since <|cosα|> = 2/π.

The effective Bijvoet ratio, is usually estimated from the following formula given by Hendrickson [15,16]:

$$\text{rms}\left({\Delta \text{F}}^{\pm }\right)/\text{rms}\left({\text{F}}_{\text{T}}\right)\approx \sqrt{{\text{N}}_{\text{A}}/2{\text{N}}_{\text{T}}}\times (2\text{f}"∕{\text{Z}}_{\text{eff}})$$

where Z_eff_ of 6.7 is the number of electrons in the “average” protein atom.

Taking into account the characteristics of the random walk and the fact that <|cosα|> = 2/π, not 1/2 assumed by Hendrickson, the formula should be modified to:

$$\text{rms}\left({\Delta \text{F}}^{\pm }\right)/\text{rms}\left({\text{F}}_{\text{T}}\right)\approx \sqrt{{\text{N}}_{\text{A}}/{\text{N}}_{\text{T}}}\times (4\text{f}"∕{\pi \text{Z}}_{\text{eff}})\approx 0.2\text{f}"\sqrt{{\text{N}}_{\text{A}}/{\text{N}}_{\text{T}}}$$

However, the situation is different if the anomalous atoms constitute the majority of the total structure, as is the case in the crystal of SeUrea, illustrated in Fig 6C. Here, the red vector F_A_ is much larger than the black vector F_N_, and it can never be perpendicular to the total vector F_T_, so that the Bijvoet difference ΔF^±^ will never reach the maximum value of 2F”. The maximum projection of 2F” onto F_T_ occurs when F_N_ is perpendicular to F_T_, and the angle γ is in maximum, with sinγ_max_ = F_N_/F_A_, and then (ΔF^±^)_max_ = 2F” sinγ_max_. The average <ΔF^±^> = 2F” ∫ sinγ dγγ from 0 to γ_max_ = 2F” [1-√(1- F_N_^2^/F_A_^2^)]. It is evident that if the structure consists of only anomalous atoms of the same kind, the anomalous Bijvoet differences vanish completely [17,18], although the measured amplitudes depend on the X-ray wavelength through the changing f’ values.

For SeUrea with F_N_^2^ = 6^2^ +2×7^2^ = 134 and F_A_^2^ = 1156, the ΔF^±^/2F” factor is equal to 0.06, meaning that the anomalous signal is diminished more than 10 times in comparison with the cases when F_A_ < F_N_, and the ΔF^±^/2F” factor is 2/π = 0.64. Nevertheless, the diffraction data measured for SeUrea contain a significant amount of anomalous signal with the averaged <ΔF^±^>/<F> = 4.5%. That value is much higher than what is available in many macromolecular diffraction data sets, where often the anomalous signal of sulfur smaller than 1% is enough to solve structures of native proteins by the SAD approach. It was, however, not possible to locate the positions of selenium atoms in the SeUrea structure from their anomalous signal by the *SHELXD* program [19], in spite of many attempts. This structure can be, of course, easily solved by direct methods from the native data.

The failure of the SAD approach is due to the fact that the measured Bijvoet differences do not correlate effectively with the amplitudes F_A_ representing diffraction of the anomalous atoms. The phase estimation in the SAD and MAD methods is based on the fact that (for the largest anomalous differences) the total reflection phases are larger by about 90° than the anomalous substructure phases. In case of SeUrea this is not true, since all substructure phases are close to the total phases, as is evident from Fig 6B. The anomalous difference map calculated in a standard way, i.e. with phases smaller by 90° from the total phases will not identify the correct positions of anomalous atoms.

## Conclusions

The high resolution diffraction data obtained for selenourea from the synchrotron source provided the structure about ten times more precise than available previously. The accurate values of bond lengths and angles as well as hydrogen bonds in SeUrea can serve as geometrical restraints used in the refinement of macromolecular structures solved with SeUrea as a phasing vehicle. The structure of SeUrea revealed a high degree of pseudosymmetry and some unusual anomalous diffraction properties. The high proportion of anomalously scattering selenium atoms in the structure precluded the use of the standard SAD phasing procedures, suggesting that too many anomalous scatterers in the structure create “embarras de richesses”, instead of making the structure solution easier. However, when SeUrea is soaked into crystals of macromolecules [5], the selenium atoms constitute only a small fraction of the total structure and their full anomalous scattering potential can be utilized for estimation of phases by the standard SAD or MAD methods.
